# Patient involvement in questionnaire design: tackling response error and burden

**DOI:** 10.1186/s40545-019-0175-0

**Published:** 2019-06-17

**Authors:** Marissa Ayano Mes, Amy Hai Yan Chan, Vari Wileman, Caroline Brigitte Katzer, Melissa Goodbourn, Steven Towndrow, Stephanie Jane Caroline Taylor, Rob Horne

**Affiliations:** 1 Centre for Behavioural Medicine, UCL School of Pharmacy, Mezzanine Floor, BMA House, Tavistock Square, London, WC1H 9JP UK; 2 Usher Institute of Population Health Sciences and Informatics, University of Edinburgh, Old Medical School, Teviot Place, Edinburgh, EH8 9AG UK; 3 Asthma UK Centre for Applied Research, Usher Institute, University of Edinburgh, Old Medical School, Teviot Place, Edinburgh, EH8 9AG UK; 4 National Institute for Health Research Collaboration for Leadership in Applied Health Research and Care North Thames, Barts Health NHS Trust, London, UK; 5 Centre for Primary Care and Public Health, Blizard Institute, Queen Mary University London, Yvonne Carter Building, 58 Turner Street, London, E1 2AB UK

**Keywords:** Patient and public involvement (PPI), Think Aloud Tasks (TATs)., Cognitive Interviewing., Questionnaire Design.

## Abstract

Questionnaires capture patient perspectives succinctly and at relatively low cost, making them a popular data collection tool for health researchers. However, questionnaire data can be affected by response error and response burden. Patient involvement during questionnaire design can help reduce the effect of response error and burden. This paper describes a novel approach for patient involvement during questionnaire design, combining methods from cognitive interviewing (Think Aloud Tasks) with an open-ended follow-up discussion to collate and act on patient feedback, while also taking account of the common challenges in questionnaire design (i.e. response error and burden). The strengths and limitations of this approach are discussed, and recommendations are made for future use.

## Plain english summary

Health researchers often use questionnaires to collect data. When patients fill in questionnaires, they may interpret the questions differently from how the researchers intended. This is an example of response error. Answers can also be affected by how much effort it takes to fill in the questionnaire, this is called response burden. For example, patients may pay less attention at the end of a long questionnaire. Involving patients in questionnaire design is important because it can help prevent response error and burden. This paper describes a new way of gathering patient feedback during questionnaire design, which combines techniques used in research with an open-ended discussion. It describes the strengths and weaknesses of this approach, and outlines tips for researchers and patients involved in questionnaire design.

## Introduction

Patient and Public Involvement (PPI) refers to “research being carried out ‘with’ or ‘by’ members of the public, rather than ‘to’, ‘about’ or ‘for’ them”. It is an integral part of healthcare policy and research guidance [1]. An example of PPI is when patient contributors provide feedback to develop new research materials such as questionnaires. Involving patients while designing new questionnaires improves questionnaire comprehensiveness and relevance, and reduces item ambiguities [2, 3].

In health research, key challenges in questionnaire design include response error and response burden [4–7]. Response error occurs when a questionnaire item or the way a respondent processes the item results in an inaccurate answer [6]. Response burden refers to the effort required to complete the questionnaire, determined by factors such as the emotional/cognitive load of items, questionnaire length, layout, or distribution method [6, 7].

In this commentary, we describe a novel approach for tackling the aforementioned challenges and facilitating PPI in questionnaire design. By drawing on methods previously used in cognitive interviewing, we aimed to identify potential sources of response error/burden and generate an initial springboard for additional discussion between researchers and patient contributors. The commentary acts as an illustrative case of health-related questionnaire design, including key insights, resources from the literature, and recommendations from the patient contributors involved during questionnaire design.

### Main text

#### Case background

We designed an intervention targeting medication adherence in adults with asthma, to be delivered by general practice pharmacists. To explore the perspectives of adults with asthma on this new intervention, we wanted to design a questionnaire that measured intervention acceptability. Acceptability refers to how appropriate patients feel an intervention is, based on experienced or anticipated cognitive/emotional responses [8].

#### Designing the questionnaire

The first version of the acceptability questionnaire was drafted by researchers based on the Theoretical Framework of Acceptability (TFA), focusing on intervention content and delivery (i.e. by pharmacists) [8]. Four members of the Asthma UK Centre for Applied Research (AUKCAR) Patient Advisory Group agreed to help refine the questionnaire as patient contributors. The AUKCAR Patient Advisory Group consists of adults with lived experience of asthma who offer advice and guidance on incorporating patient perspectives into all stages of research.

Feedback was gathered over the telephone, enabling contributors from across the United Kingdom (UK) to be involved. The telephone calls were audio-recorded for our records and detailed summaries of patient feedback were written. Drawing on cognitive interviewing methodology, we began with a Think Aloud Task (TAT) based on recommendations by van Someren MW, Barnard YF and Sandberg JAC [9]. Contributors were asked to continuously verbalise their thought process as they worked through the questionnaire. This helped researchers understand how people interpreted, processed, and responded to questionnaire items [9]. Researchers and contributors then picked up on issues emerging from the TATs in an open-ended follow-up discussion, where contributors were also encouraged to bring up any additional issues they found relevant. Patient contributors were e-mailed TAT instructions and background information on the intervention ahead of time. To ensure that contributors felt comfortable with the TAT procedure, an unrelated questionnaire about job satisfaction was used to practice. We did not e-mail the acceptability questionnaire to the contributors until directly before the telephone call to ensure that the TAT was based on their first impressions. Patient contributors’ feedback was incorporated into a new questionnaire draft (see Table 1 for examples). To keep patient contributors informed, we produced a brief summary of their feedback and how it was incorporated into the new questionnaire. This was e-mailed to them, in case of further comments.

**Table 1:** Examples of patient contributors’ feedback and the associated changes to the questionnaire

Identified in	Example of	Feedback given	Changes made
TAT	Response error	In the medication adherence literature, “concerns” refer to medication-related negative effects [10]. However, patient contributors interpreted the item “I am concerned about my preventer inhaler” as “I care about my preventer inhaler”.	The item wording was changed to “I am worried about taking my preventer inhaler”.
Follow-up discussion	External factors	The questionnaire was designed for completion in a general practice waiting room. While patient contributors felt that the instructions on the questionnaire were clear, they noted that elements of the waiting rooms (e.g. ambient noise or waiting to be called in) may distract respondents from reading the instructions thoroughly.	The questionnaire instructions were shortened and illustrations were added to provide crucial information in a single glance.

### Key insights and recommendations

Two patient contributors reflected on the questionnaire design process with the researchers to identify the strengths and limitations of our novel PPI approach (TATs paired with an open-ended discussion). Recommendations for this approach are highlighted in Table 2.

**Table 2:** Key recommendations from researchers and patient contributors about the novel approach (TATs and follow-up discussion) for patient involvement in questionnaire design

Key Recommendations	Description
Clear TAT instructions	• Ask contributors to continuously say what is going through their minds with limited pauses. • Avoid asking for evaluation (e.g. “tell me what you think about…”). • Always ask if contributors have any questions about the TAT procedure.
Practice TAT	• Practice TATs with another questionnaire (similar structure, different topic). • Jointly reflect on the practice exercise with the contributor. • Aim for minimal researcher interruptions during the practice task.
Multiple feedback methods	• Contributors may not feel comfortable with certain feedback methods (e.g. TATs). • When using TATs, always have alternative feedback methods available (e.g. open-ended feedback sessions). • Use structured feedback methods (e.g. TATs) as a springboard for other feedback methods (e.g. open-ended discussion).
Good rapport	• Establish good researcher – contributor rapport before gathering feedback. • Making contributors feel comfortable will generate more detailed feedback. • Making sure contributors have a positive PPI experience will benefit future research (e.g. with their continued involvement in other studies).

A key strength of the TAT is the fact that it structures the initial feedback process. Patient contributors mentioned that this may be a useful approach for people who initially struggle with open-ended questions. The TAT also facilitated researcher and contributor communication by acting as a springboard for the open-ended follow-up discussion: researchers picked up on issues identified in the TAT for further discussion with the contributors.

However, a potential limitation of the approach is that contributors may have limited experience with TATs, and this may act as a barrier to feedback. We followed recommendations made by van Someren MW, Barnard YF and Sandberg JAC [9] regarding clear TAT instructions: asking contributors to continuously say what was going through their minds with limited pauses, rather than asking for evaluation (e.g. “what do you think about…”). A practice TAT with an unrelated questionnaire also helped contributors familiarise themselves with the procedure [9]. We tried to overcome the limitations associated with the TATs with an open-ended follow-up discussion, which tackled issues that had not been identified in the TAT procedure (e.g. external factors) (see Table 1). Furthermore, employing multiple feedback methods ensured that contributors were able to offer feedback in a way that was comfortable for them.

Underpinning this entire process was the rapport between researchers and patient contributors. Contributors emphasized the importance of investing adequate time to establish rapport before gathering feedback. Contributors that feel comfortable with researchers may provide more detailed feedback, and giving contributors a positive PPI experience may encourage future research involvement. Minimising researcher interruption during both the TAT and the follow-up discussion signalled to the contributors that their time to speak was valued and respected.

## Conclusion

As well as tackling common issues in questionnaire design (e.g. response error), the novel approach outlined in this commentary also incorporated people’s lived experiences of a long-term condition and potential study settings (e.g. general practice waiting rooms) into the questionnaire design process. Using methods previously employed in cognitive interviewing, researchers and patient contributors were able to create an initial springboard for further discussion. This unique combination of feedback methods may help researchers collate and act on patient feedback, while also taking account of the common challenges in questionnaire design (i.e. response error and burden). For patient contributors involved in questionnaire design, having a clear-cut process for feedback may also improve the PPI experience. These benefits may encourage increased PPI in questionnaire design, and reduce the risk of making questionnaires overly complex.

## Abbreviations

AUKCAR: Asthma UK Centre for Applied Research
PPI: Patient and Public Involvement
TAT: Think Aloud Task
TFA: Theoretical Framework of Acceptability
UK: United Kingdom
